# Supplementary material for: Regulation of kinesin-2 motility by its β-hairpin motif
Source: Nat Struct Mol Biol. 2025 Jul 29;32(10):1989–98. doi: 10.1038/s41594-025-01630-5 (PMC12527917; doi:10.1038/s41594-025-01630-5)
Supplement: Supplementary file 1 — Reporting Summary [file 41594_2025_1630_MOESM1_ESM.pdf]

nature portfolio

Corresponding author(s): Anthony Roberts

Last updated by author(s): 23/05/25

Reporting Summary

Nature Portfolio wishes to improve the reproducibility of the work that we publish. This form provides structure for consistency and transparency in reporting. For further information on Nature Portfolio policies, see our Editorial Policies and the Editorial Policy Checklist.

Statistics

For all statistical analyses, confirm that the following items are present in the figure legend, table legend, main text, or Methods section.

n/a	Confirmed	
☐	☒	The exact sample size (n) for each experimental group/condition, given as a discrete number and unit of measurement
☐	☒	A statement on whether measurements were taken from distinct samples or whether the same sample was measured repeatedly
☐	☒	The statistical test(s) used AND whether they are one- or two-sided<br>*Only common tests should be described solely by name; describe more complex techniques in the Methods section.*
☒	☐	A description of all covariates tested
☒	☐	A description of any assumptions or corrections, such as tests of normality and adjustment for multiple comparisons
☐	☒	A full description of the statistical parameters including central tendency (e.g. means) or other basic estimates (e.g. regression coefficient) AND variation (e.g. standard deviation) or associated estimates of uncertainty (e.g. confidence intervals)
☐	☒	For null hypothesis testing, the test statistic (e.g. F, t, r) with confidence intervals, effect sizes, degrees of freedom and P value noted<br>*Give P values as exact values whenever suitable.*
☒	☐	For Bayesian analysis, information on the choice of priors and Markov chain Monte Carlo settings
☒	☐	For hierarchical and complex designs, identification of the appropriate level for tests and full reporting of outcomes
☒	☐	Estimates of effect sizes (e.g. Cohen's d, Pearson's r), indicating how they were calculated

Our web collection on statistics for biologists contains articles on many of the points above.

Software and code

Policy information about availability of computer code

Data collection	Total internal reflelection fluorescence microscopy data were collected with NIS-Elements AR software v4.60.00 (Nikon).<br>Mass photometry data were collected with AcquireMP v2 (Refeyn) and processed with DiscoverMP v2 (Refeyn) software.<br>Electron microscopy data were collected using TUI software 2017 (ThermoFisher Scientific) and DigitalMicrograph software 1.85.1535 (Gatan).
Data analysis	SPIDER v22.10, EMAN2 v2.91, Cryosparc v4.5.1, UCSF Chimera v1.16, UCSF ChimeraX v1.8, Fiji v2.9.0, GraphPad Prism v9 and v10.

For manuscripts utilizing custom algorithms or software that are central to the research but not yet described in published literature, software must be made available to editors and reviewers. We strongly encourage code deposition in a community repository (e.g. GitHub). See the Nature Portfolio guidelines for submitting code & software for further information.

Data

Policy information about availability of data

All manuscripts must include a data availability statement. This statement should provide the following information, where applicable:

- Accession codes, unique identifiers, or web links for publicly available datasets
- A description of any restrictions on data availability
- For clinical datasets or third party data, please ensure that the statement adheres to our policy

All data supporting the conclusions of this manuscript are available from the corresponding author upon reasonable request.

Research involving human participants, their data, or biological material

Policy information about studies with human participants or human data. See also policy information about sex, gender (identity/presentation), and sexual orientation and race, ethnicity and racism.

Reporting on sex and gender	NA
Reporting on race, ethnicity, or other socially relevant groupings	NA
Population characteristics	NA
Recruitment	NA
Ethics oversight	NA

Note that full information on the approval of the study protocol must also be provided in the manuscript.

Field-specific reporting

Please select the one below that is the best fit for your research. If you are not sure, read the appropriate sections before making your selection.

☒ Life sciences ☐ Behavioural & social sciences ☐ Ecological, evolutionary & environmental sciences

For a reference copy of the document with all sections, see nature.com/documents/nr-reporting-summary-flat.pdf

Life sciences study design

All studies must disclose on these points even when the disclosure is negative.

Sample size	No statistical methods were used to predetermine sample size. The number of motile events analyzed per construct was based on previous publications and standards in the field. The number of motile events recorded per movie not readily predictable in advance of the experiment.
Data exclusions	For all single molecule measurements events longer than 4 consecutive pixels were included in the analysis. Microtubules not fully enclosed in the field of view or overlapping with another microtubule were excluded from the analysis.
Replication	Experiments were carried out as technical replicates, n=3. All attempts were successful.
Randomization	Datasets were not randomized. No group allocation was performed for any experiments.
Blinding	Blinding was not used. No group allocation was performed for any experiments.

Reporting for specific materials, systems and methods

We require information from authors about some types of materials, experimental systems and methods used in many studies. Here, indicate whether each material, system or method listed is relevant to your study. If you are not sure if a list item applies to your research, read the appropriate section before selecting a response.

Materials & experimental systems

n/a	Involved in the study	
☐	☒	Antibodies
☐	☒	Eukaryotic cell lines
☒	☐	Palaeontology and archaeology
☒	☐	Animals and other organisms
☒	☐	Clinical data
☒	☐	Dual use research of concern
☒	☐	Plants

Methods

n/a	Involved in the study	
☒	☐	ChIP-seq
☒	☐	Flow cytometry
☒	☐	MRI-based neuroimaging

Antibodies

Antibodies used	Primary antibodies: ANTI-FLAG® M2 mouse antibody (1:1000; Sigma- Aldrich F1804), anti-GAPDH (1:10,000; Cell Signaling Technology 2118), anti-acetylated tubulin (1:2000; Sigma-Aldrich T6793), anti-gamma-tubulin (1:5000; Sigma-Aldrich T6557).

	Secondary antibodies: Goat Anti-Mouse IgG StarBright Blue 700 (1:1000; BioRad 12004159), Goat Anti-Rabbit IgG (H + L) Alexa Fluor 647 (1:1000; Invitrogen A-21244).
Validation	Purity, specificity and sensitivity tests carried out by manufacturer. Antibodies widely used in the field.<br>ANTI-FLAG® M2 mouse antibody: two major bands with purity >90% when analyzed by microfluidic gel capillary electrophoresis; detects a single band of protein on a Western Blot from mammalian crude cell lysates; detects 2 ng of FLAG-BAP fusion protein by Dot Blot using Chemiluminescent Detection.<br>anti-GAPDH: GAPDH (14C10) Rabbit mAb detects endogenous levels of total GAPDH protein.

Eukaryotic cell lines

Policy information about cell lines and Sex and Gender in Research

Cell line source(s)	Mouse IMCD-3-FlpIn cells (gift from Peter K. Jackson, Stanford; Mukhopadhyay et al (2010) Genes Dev. 24, 2180–2193). Sf9 cells (Gibco 11496015).
Authentication	Cell lines were not authenticated.
Mycoplasma contamination	Parental cell lines were verified to be free from mycoplasma using a PCR detection kit (Sigma-Aldrich, MP0035).
Commonly misidentified lines<br>(See ICLAC register)	No commonly misidentified lines used.

Plants

Seed stocks	*Report on the source of all seed stocks or other plant material used. If applicable, state the seed stock centre and catalogue number. If plant specimens were collected from the field, describe the collection location, date and sampling procedures.*
Novel plant genotypes	*Describe the methods by which all novel plant genotypes were produced. This includes those generated by transgenic approaches, gene editing, chemical/radiation-based mutagenesis and hybridization. For transgenic lines, describe the transformation method, the number of independent lines analyzed and the generation upon which experiments were performed. For gene-edited lines, describe the editor used, the endogenous sequence targeted for editing, the targeting guide RNA sequence (if applicable) and how the editor was applied.*
Authentication	*Describe any authentication procedures for each seed stock used or novel genotype generated. Describe any experiments used to assess the effect of a mutation and, where applicable, how potential secondary effects (e.g. second site T-DNA insertions, mosiacism, off-target gene editing) were examined.*
