# Supplementary material for: Regulation of kinesin-2 motility by its β-hairpin motif
Source: Nat Struct Mol Biol. 2025 Jul 29;32(10):1989–98. doi: 10.1038/s41594-025-01630-5 (PMC12527917; doi:10.1038/s41594-025-01630-5)

Gel and western blot source data, Figure 1

Figure 1f, left

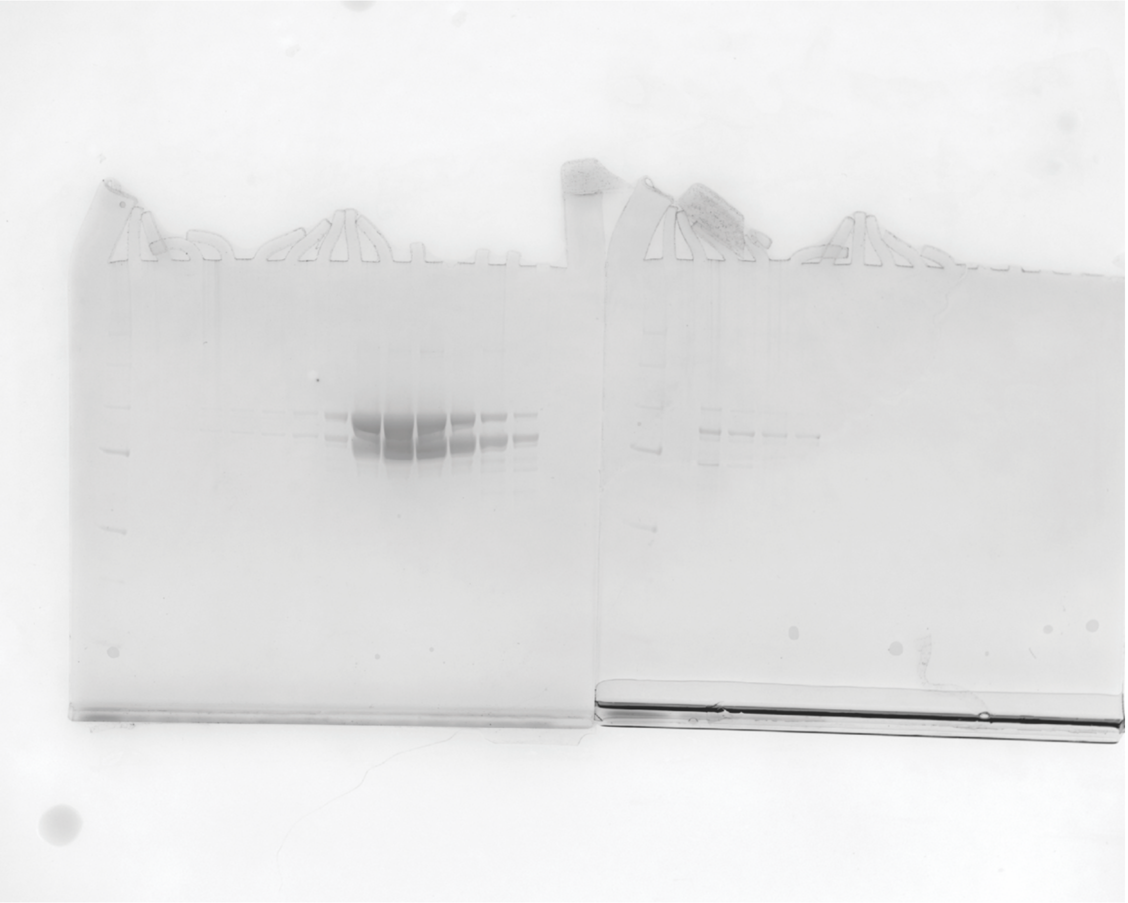

Figure 1f, right

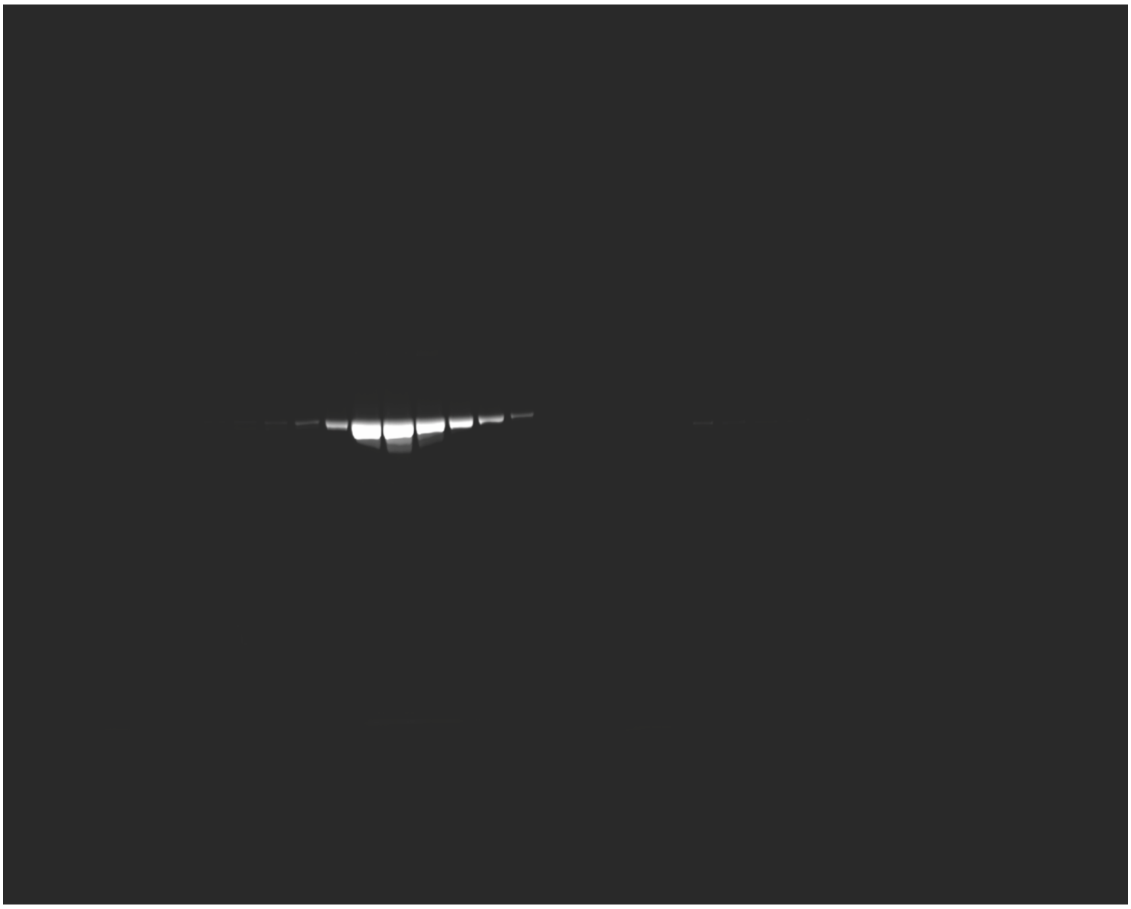

Gel and western blot source data, Figure 4

Figure 4b

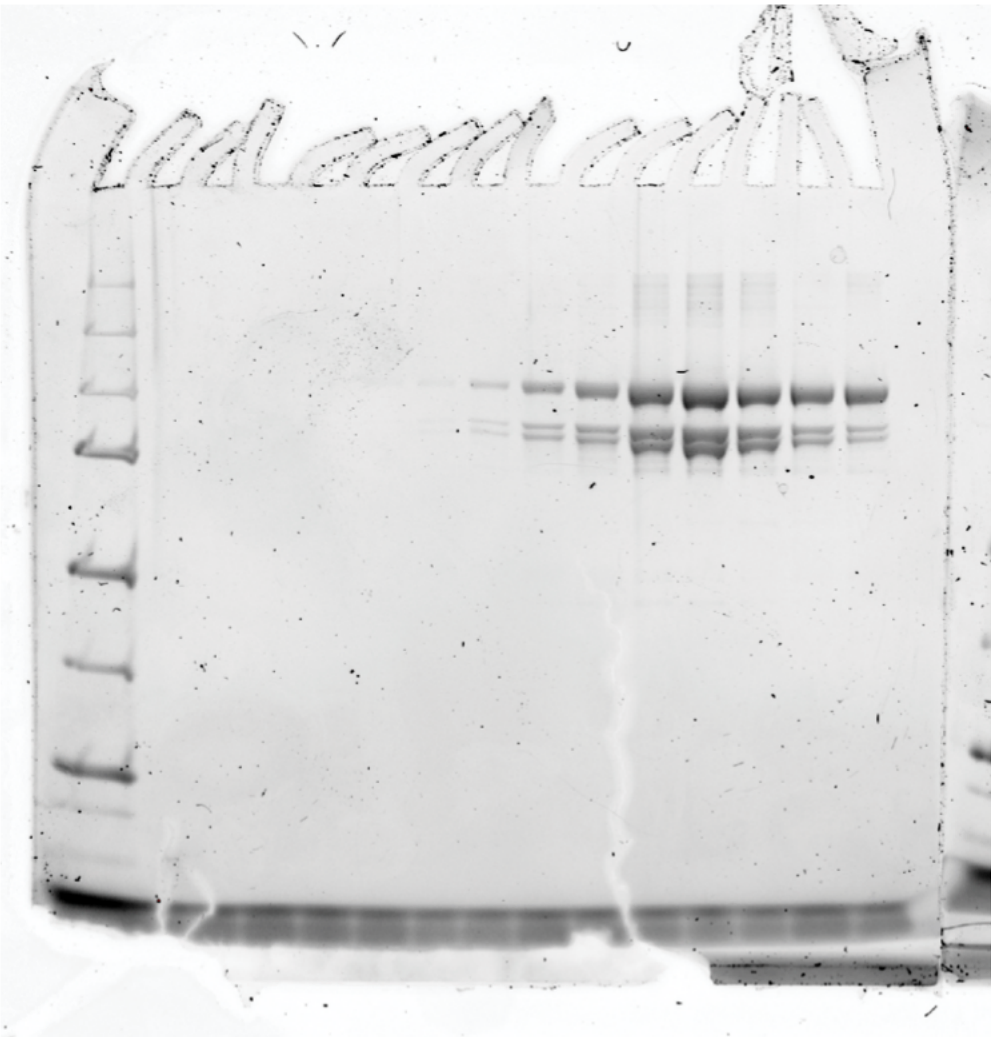

Gel and western blot source data, Figure 5

Figure 5e

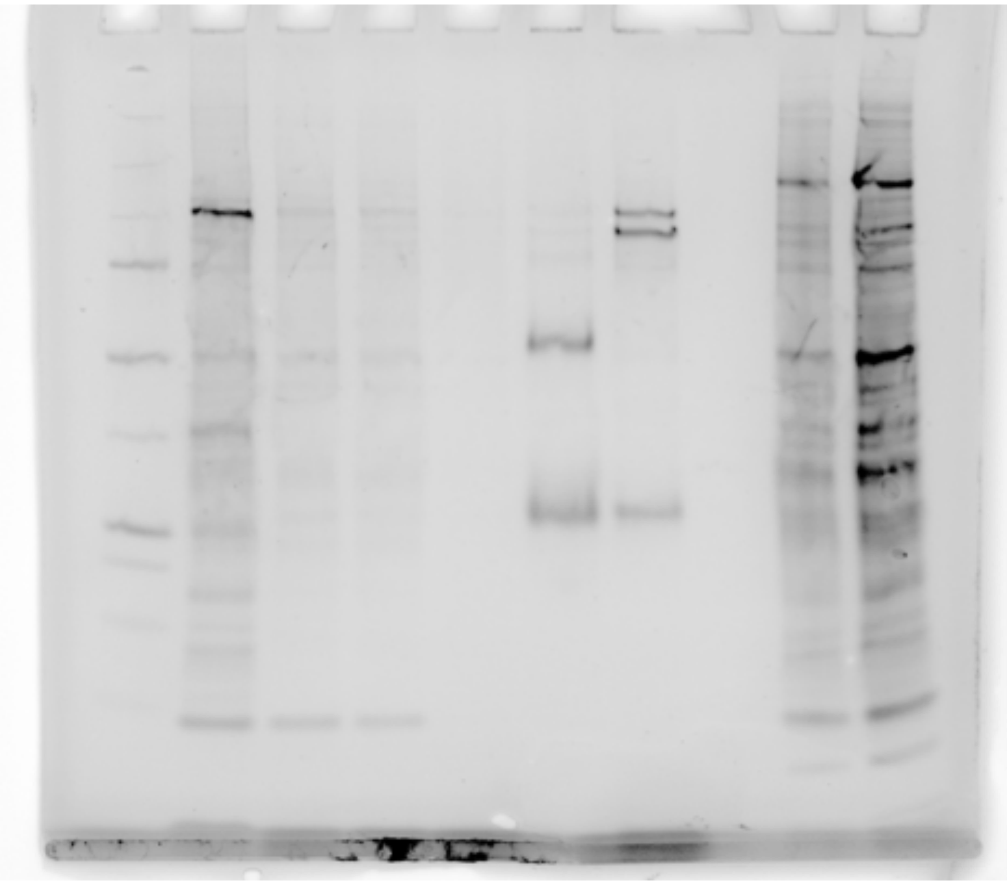

Gel and western blot source data, Figure 6

Figure 6a

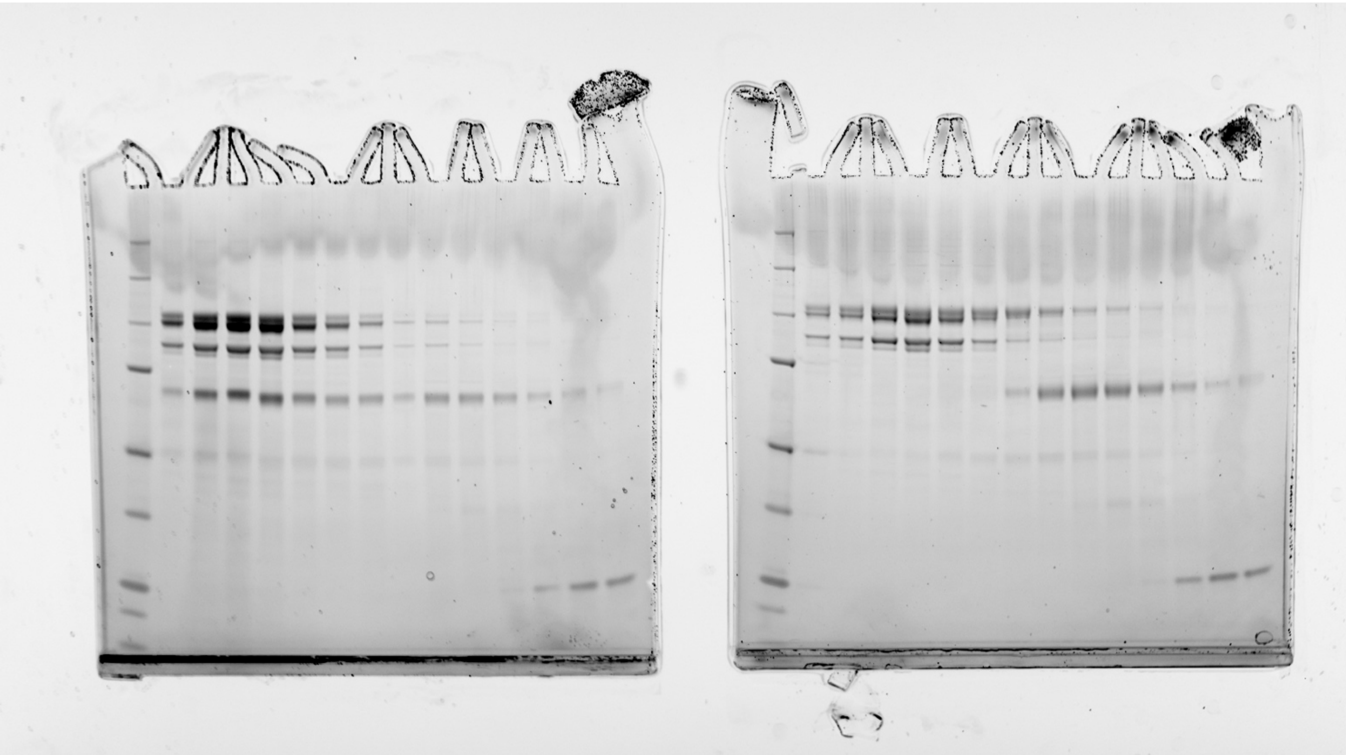

Gel and western blot source data, Extended Data Figure 2

Extended Data Figure 2a

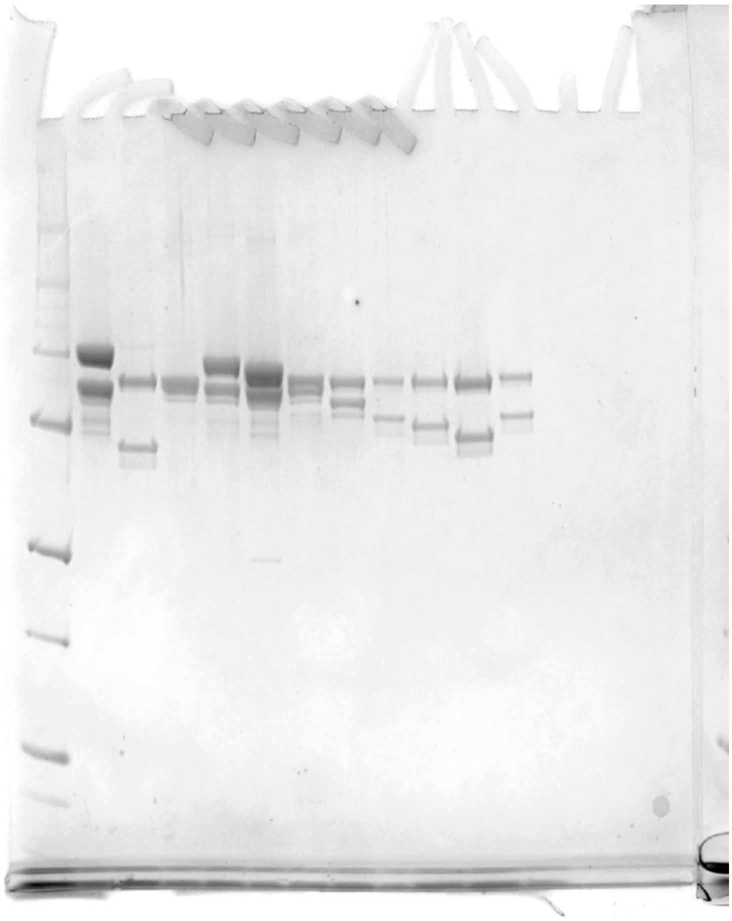

Extended Data Figure 2d, upper

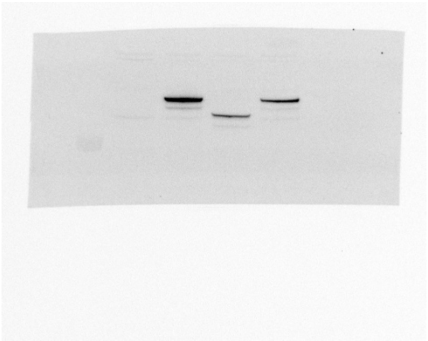

Extended Data Figure 2d, lower

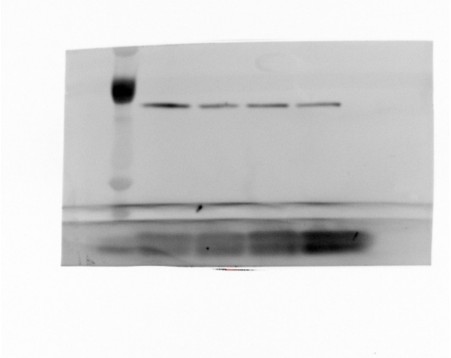

Gel and western blot source data, Extended Data Figure 3

Extended Data Figure 3d

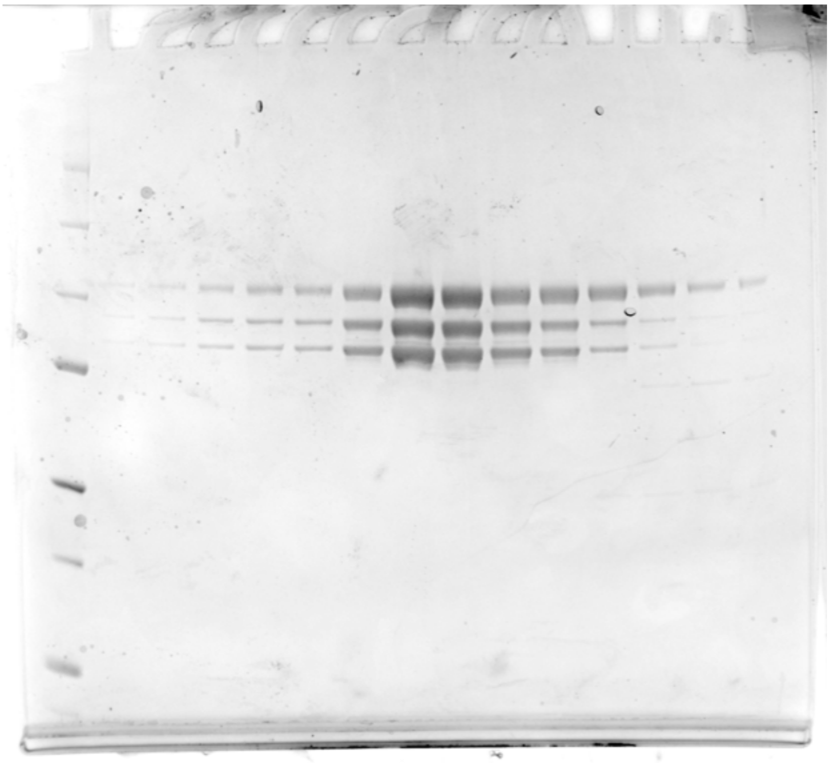

Gel and western blot source data, Extended Data Figure 4

Extended Data Figure 4a, left

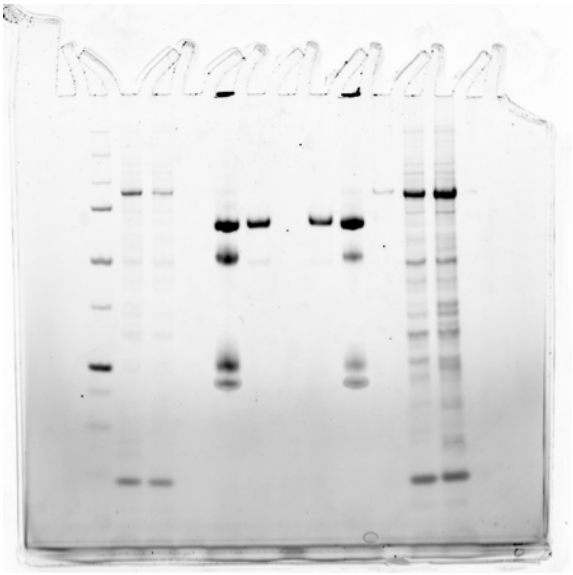

Extended Data Figure 4a, right

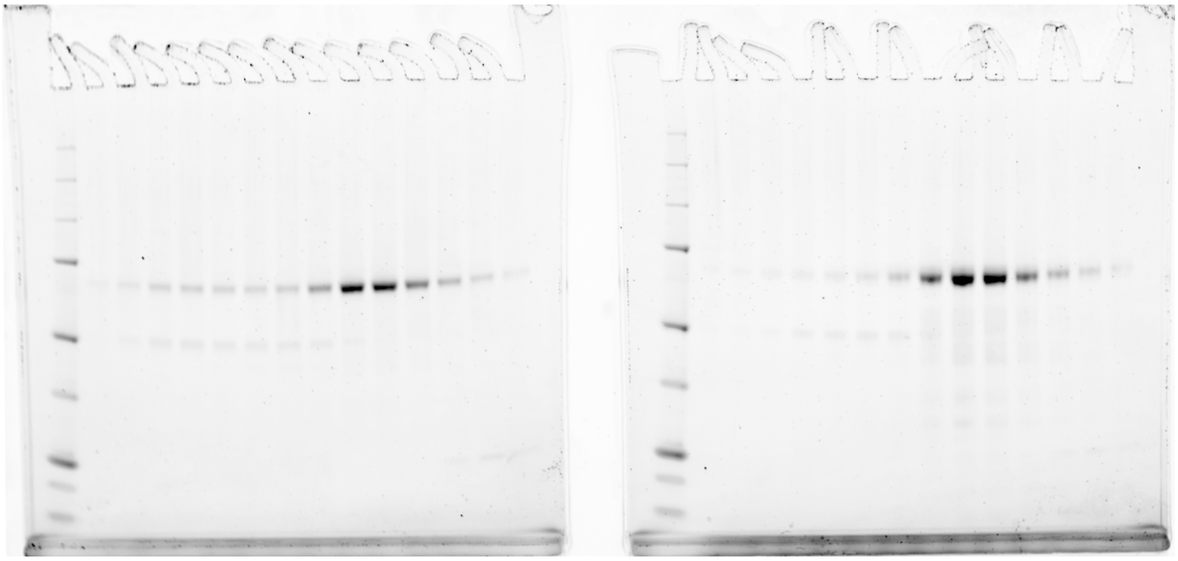

Extended Data Figure 4b

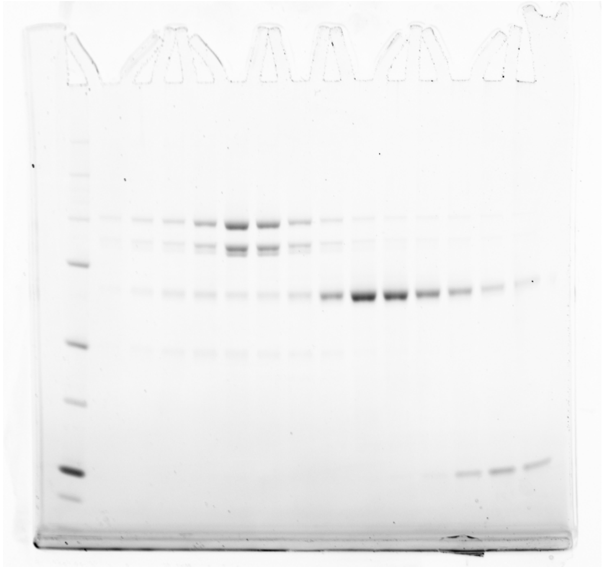

Supplement: Supplementary file 4 — Unprocessed gels. [file 41594_2025_1630_MOESM4_ESM.pdf]
